# Partial disconnection procedure in a patient with bilateral lesions (case report)^☆^

**DOI:** 10.1016/j.ebcr.2013.02.002

**Published:** 2013-03-29

**Authors:** Alexey Y. Stepanenko, Natalia A. Arkhipova, Igor N. Pronin, Lyudmila V. Shishkina, Anna V. Lebedeva, Alla B. Guekht

**Affiliations:** a Department of Neurosurgery, Moscow City Hospital No. 12, 26 Bakinskaya Street, Moscow 115516, Russian Federation; b N. N. Burdenko Neurosurgery Institute, 16 4th Tverskaya-Yamskaya Street, Moscow 125047, Russian Federation; c Department of Neurology and Neurosurgery, Russian State Research Medical University, 8, Block 8 Leninsky Prospect, Moscow 119049, Russian Federation; d Department of Neurology, Moscow City Hospital No. 12, 26 Bakinskaya Street, Moscow 115516, Russian Federation; e Moscow City Hospital No. 8, 43 Donskaya Street, Moscow 115419, Russian Federation

**Keywords:** Epilepsy surgery, Partial disconnection procedure, Cortical dysplasia, Nodular heterotopias

## Abstract

**Purpose:**

The method of temporal lobectomy and parietooccipital disconnection has been applied in the treatment of patients with monolateral widespread cortical lesions and with hand motor function intact. There are no data regarding the use of this method in the treatment of patients with bilateral lesions.

**Case report:**

A case history of a 15-year-old female patient with medically refractory epilepsy is presented. Magnetic resonance imaging revealed bilateral periventricular nodular heterotopia associated with cortical dysplasia (CD) in the right temporo-parietal region. The left hemisphere had no signs of CD. Invasive monitoring revealed rhythmic theta–delta activity during the interictal period and fast activity during the ictal onset in the right temporal and parietal regions. The surgery procedure consisted of anterior temporal lobectomy, the removal of the right heterotopy nodus, the dissection of the posterior part of the corpus callosum, and the detachment of the temporo-parieto-occipital complex by dissection behind the sensorimotor cortex. Histological examination of the cortex revealed CD type I. The patient has been seizure-free for 4 years after surgery.

**Conclusion:**

Partial disconnection procedures may be effective in cases where total hemispherotomy is not indicated in patients with bilateral lesions and a well-lateralized epileptogenic zone localized in the temporo-parieto-occipital region.

## 1 Introduction

Malformations due to abnormal cortical development are common pathologic substrates of medically intractable epilepsy. Some varieties are intrinsically epileptogenic; these include cortical dysplasia (CD) and nodular heterotopia (NH). Cortical dysplasia type I is characterized by frequent multilobar involvement and negative or nonspecific MRI imaging, and these are the most important factors of poor prognosis of surgical treatment [1], [2], [3], [4]. Nodular heterotopia frequently manifests as bilateral nodules, sometimes associated with other brain malformations [5], [6]. Generally, bilateral lesions are considered as contraindications to resective or disconnection surgical procedures.

The so-called “hemi-hemispherotomy” was suggested by T. Rasmussen [7] and later, under the name of temporal lobectomy and posterior (parietooccipital) disconnection, it was applied in the treatment of patients with monolateral widespread cortical lesions localized in temporo-parietal regions and with hand motor function intact [8]. There are no data regarding the use of this method in the treatment of patients with bilateral lesions.

## 2 Case report

**Fig. 1:**
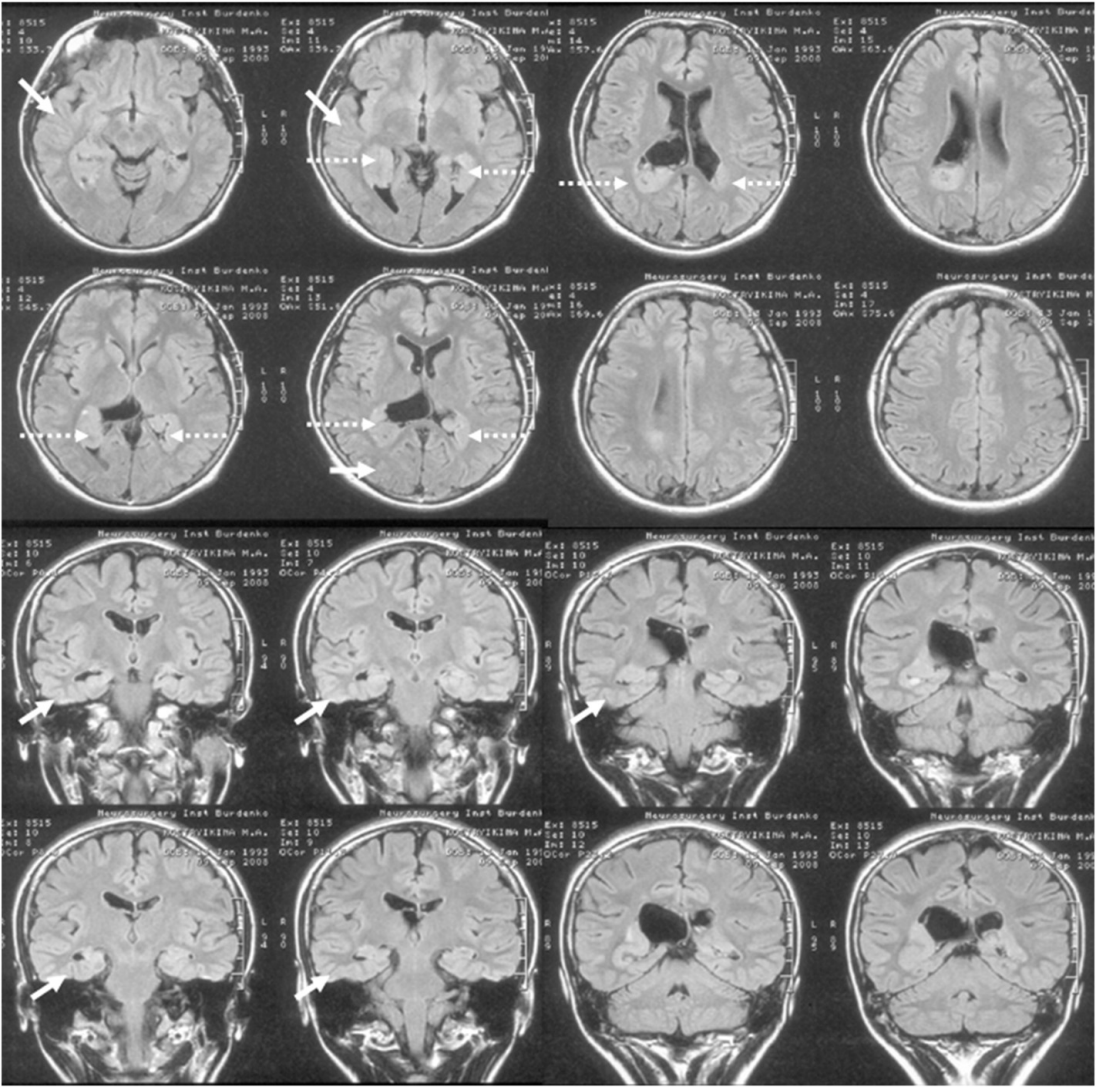
Magnetic resonance imaging findings in the patient: regions of cortical dysplasia (arrows); nodules of heterotopic matter (interrupted arrows).

A 15-year-old female patient with medically refractory epilepsy was treated surgically in N. N. Burdenko Neurosurgery Institute, Moscow, in October 2008. The patient had no family history of epilepsy. Pregnancy and delivery were reported as normal. First seizures occurred at the age of 9. The patient suffered from pharmacoresistant complex partial seizures without aura appearing with loss of consciousness, aversion to the right, falling, and sometimes with secondary generalization, with a frequency of 2–4 seizures per month, without any response to antiepileptic drug therapy. Therapy on admission was as follows: valproate — 2000 mg per day and carbamazepine — 1000 mg per day. She had no neurological deficit, and she was able to attend a regular school. Magnetic resonance imaging revealed bilateral malformation of cortical development and bilateral periventricular NT associated with CD in the right temporo-parietal region; the latter appeared in slight MRI abnormalities such as thickening of the cortex and dysgyria, which could be considered as CD. Mild atrophy of the right hemisphere was also noted (Fig. 1). The left hemisphere had no signs of CD. Surface EEG recording showed bilateral interictal epileptic discharges, without any focal seizure onset.

At first, the patient was considered as an unfavorable candidate for surgery because of bilateral lesions, but in view of the health hazard of unexpected falling during seizures, the decision was later made to continue presurgical evaluation and to make invasive neurophysiological investigation.

**Fig. 2:**
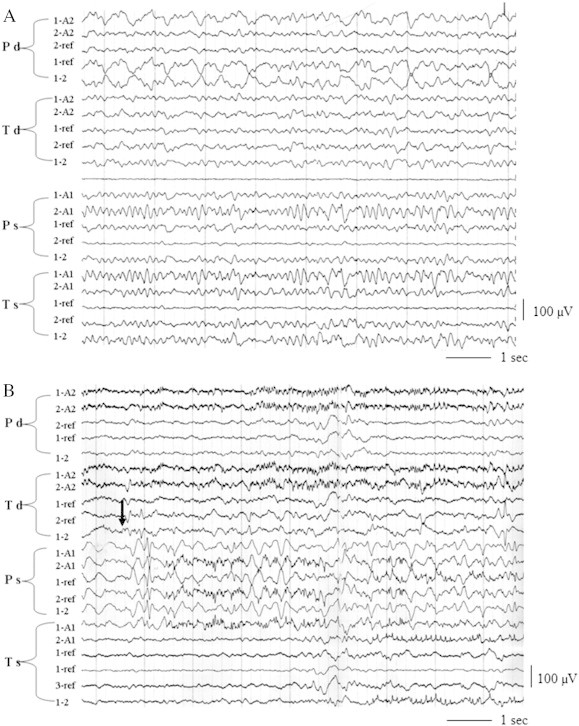
Neurophysiological findings (invasive recording). (A) Interictal intermitted delta activity in the medial part of the right parietal lobe. Disorganization of background activity in the medial part of the right temporal lobe. (B) Ictal activity: low-amplitude fast activity in the medial part of the right temporal lobe (Td, 1–2 — arrow) and the following sharp-wave activity in the medial part of the left parietal lobe and depression of background activity in the medial part of the left temporal lobe.

Invasive monitoring was performed with the use of intra-cerebral electrodes placed according to stereotactic methods in the medial temporal and parietal regions and nodules of heterotopia on both sides. There was a marked disorganization of background activity with periods of rhythmic theta–delta activity in the medial temporal and parietal regions on the right side during the interictal period. Ictal onset was characterized by the appearance of fast activity in the right medial temporal regions followed by the rapid spreading of epileptic discharge to the left hemisphere (Fig. 2).

Based on the neurophysiological data, the right temporal and parietal cortices were considered as part of the epileptogenic zone, but we supposed that the real dimension of the epileptogenic zone was more extensive than only the right temporal and parietal cortices. It was entirely possible that the left-side NH would be epileptogenic too. Nevertheless, we took the decision to perform a right-side partial disconnection procedure expecting a palliative result — the prevention of secondary generalization of seizures. The surgery was performed in December 2008.

**Fig. 3:**
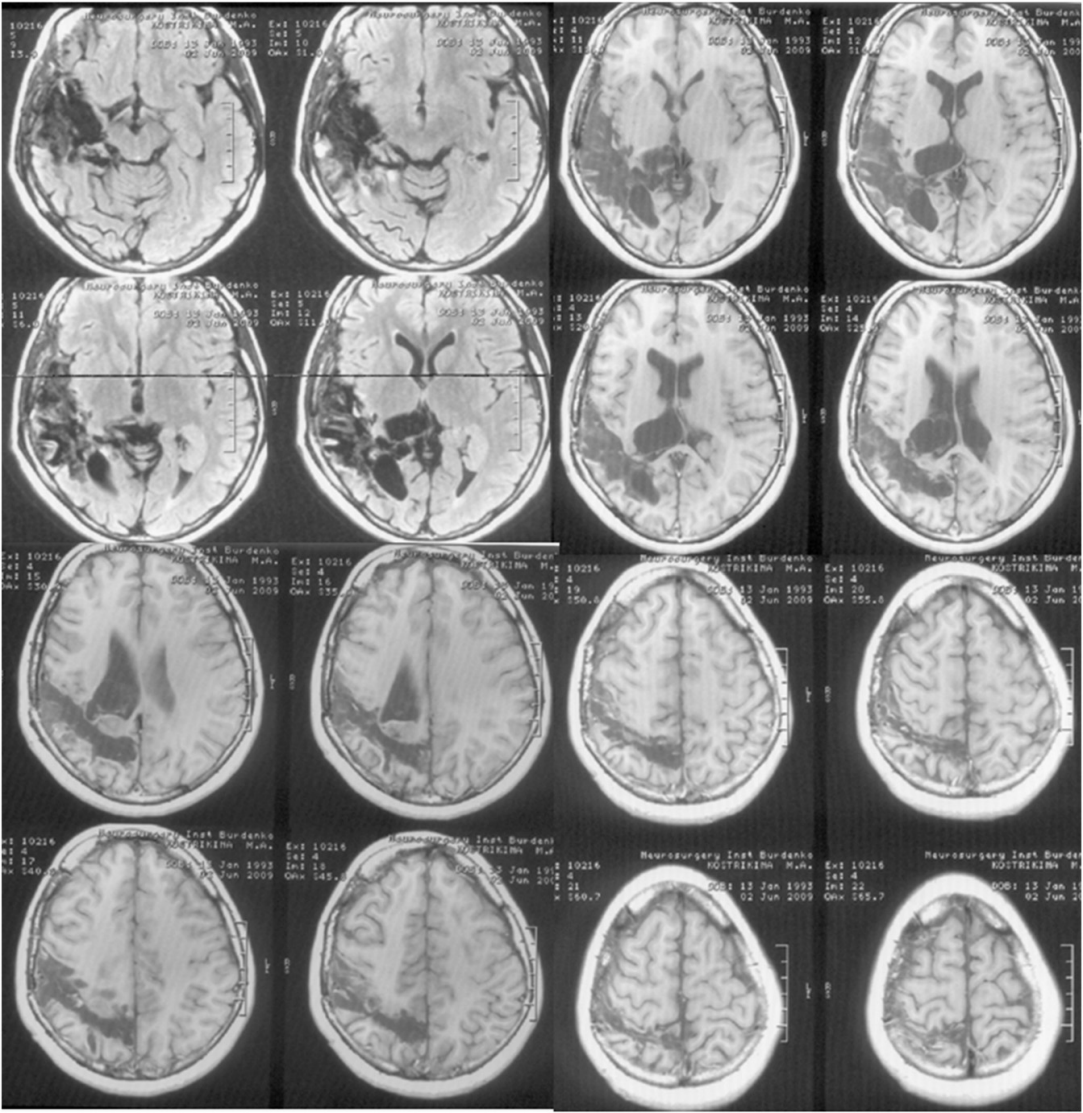
Postoperative MRI. Volume of surgical intervention (described in the text of the article).

The surgery procedure consisted of anterior temporal lobectomy and the opening of the posterior part of the temporal horn by dissection through the medial margin of the superior temporal gyrus to the trigonum, the removal of the right heterotopy nodus, the dissection of the parahippocampal gyrus and the medial part of the parietal lobe at the level of the posterior part of the ambiens cistern, the dissection of the posterior part of the corpus callosum, and the detachment of the temporo-parieto-occipital complex by dissection through the white matter of the parietal lobe just behind the sensorimotor cortex (Fig. 3).

Histological examination of the basal temporal and parietal cortices and the hippocampus revealed CD type I according to the ILAE classification [4] with immature neurons — type 1B according to the Palmini classification [2].

The patient was noted to have permanent hemianopia and transient hemiparesis for the first 2 months after surgery with complete recovery.

Standardized follow-up evaluations were performed every 6 months after surgery. The patient has been seizure-free for more than 4 years after surgery and was treated with the same therapy as that used before surgery. She goes to college now.

## 3 Discussion

In this study, we report a case of bilateral lesions with MRI evidence supported by histology, with the patient having been operated on. The presence of bilateral lesions is often considered as a contraindication to resective surgery, but it should not be considered so in all cases. The case shows a different epileptogenic potential of malformation because of abnormal cortical development. Patients with neuronal migration disorders often show simultaneous ictal onset within the periventricular NH and the overlying cortex. These patients become seizure-free after the resection of the nodular tissue and the corresponding dysplastic cortex [5]. In some cases, heterotopic nodules can generate normal activity and may not be epileptogenic [9]. Furthermore, experimental data do not support the concept that NH lesions are the primary epileptogenic but rather point to the overlying cortex, which has been shown in a rat model [10]. Perhaps, the epileptogenicity of the dysplastic cortex is the main condition for generating epileptic activity in the cortex-NH functional complexes.

Based on the literature data, we suppose that the association of cortical dysplasia and heterotopy is more epileptogenic than heterotopy alone. In our case, the left-side lesion was evidently less epileptogenic, and its epileptogenic potential did not appear on the anticonvulsant therapy. Partial disconnection procedures may be effective in cases where total hemispherotomy is not indicated and also in patients with bilateral lesions and well-lateralized ictal onset and irritative zones localized in the temporo-parieto-occipital region.

## Conflict of interest

There is no actual or potential financial and other conflict of interest related to the submitted manuscript.
